# Total Outflow of High-Density Lipoprotein–Cholesteryl Esters from Plasma Is Decreased in a Model of 3/4 Renal Mass Reduction

**DOI:** 10.3390/ijms242317090

**Published:** 2023-12-04

**Authors:** María Luna-Luna, Martha Franco, Elizabeth Carreón-Torres, Nonanzit Pérez-Hernández, José Manuel Fragoso, Rocío Bautista-Pérez, Óscar Pérez-Méndez

**Affiliations:** 1 Department of Molecular Biology, Instituto Nacional de Cardiologia “Ignacio Chavez”, Mexico City 14080, Mexico; mjluna.qfb@gmail.com (M.L.-L.); elizact73@gmail.com (E.C.-T.); unicanona@yahoo.com.mx (N.P.-H.); mfragoso1275@yahoo.com.mx (J.M.F.); rociobtst@yahoo.com (R.B.-P.); 2 Department of Nephrology, Instituto Nacional de Cardiologia “Ignacio Chavez”, Mexico City 14080, Mexico; marthafranco@lycos.com; 3 Tecnologico de Monterrey, Campus Ciudad de Mexico, Mexico City 14380, Mexico

**Keywords:** cholesteryl ester, lipoprotein metabolism, renal damage

## Abstract

(1) Background: Previous studies have enriched high-density lipoproteins (HDL) using cholesteryl esters in rabbits with a three-quarter reduction in functional renal mass, suggesting that the kidneys participate in the cholesterol homeostasis of these lipoproteins. However, the possible role of the kidneys in lipoprotein metabolism is still controversial. To understand the role of the kidneys in regulating the HDL lipid content, we determined the turnover of HDL-cholesteryl esters in rabbits with a three-quarter renal mass reduction. (2) Methods: HDL subclass characterization was conducted, and the kinetics of plasma HDL-cholesteryl esters, labeled with tritium, were studied in rabbits with a 75% reduction in functional renal mass (Ntx). (3) Results: The reduced renal mass triggered the enrichment of cholesterol, specifically cholesteryl esters, in HDL subclasses. The exchange of cholesteryl esters between HDL and apo B-containing lipoproteins (VLDL/LDL) was not significantly modified in Ntx rabbits. Moreover, the cholesteryl esters of HDL and VLDL/LDL fluxes from the plasmatic compartment tended to decrease, but they only reached statistical significance when both fluxes were added to the Nxt group. Accordingly, the fractional catabolic rate (FCR) of the HDL-cholesteryl esters was lower in Ntx rabbits, concomitantly with its accumulation in HDL subclasses, probably because of the reduced mass of renal cells requiring this lipid from lipoproteins.

## 1. Introduction

The well-known negative correlation between HDL-cholesterol (HDL-C) and the development of coronary heart disease suggests a causal relationship [1,2]; this is explained by reverse cholesterol transport (RCT), in which HDL uptake cholesterol from foam cells and reduce their lipid content, thus delaying atheroma formation [3]. Furthermore, other potentially anti-atherogenic properties of HDL have been demonstrated, such as (1) their anti-inflammatory capacity, inhibiting the expression of endothelial adhesion molecules, including vascular cell adhesion molecule-1, intercellular adhesion molecule-1, P-selectin, and E-selectin [4,5,6,7,8]; (2) anti-thrombotic effects, attenuating the tissue factor, thrombin, and the soluble CD40L-CD40 system [3,8]; (3) the presence of apo A1, lecithin–cholesterol acyltransferase, paraoxonase-1, and platelet-activating factor acetylhydrolase, which confer anti-oxidative properties upon HDL [4,9]; and (4) the improvement in endothelial function after inducing the activity of endothelial NO synthase [5,10]. Based on these potential anti-atherogenic properties, particularly the RCT paradigm, several pharmacological interventions have been developed; however, they have failed to reduce the cardiovascular risk [11,12,13], indicating that the physiological role of these lipoproteins is not yet fully understood.

HDL comprise a heterogeneous group of lipoproteins that may be classified by decreasing size as HDL 2b, HDL 2a, HDL 3a, HDL 3b, and HDL 3c [14]. These subclasses are generated during the intravascular metabolism of HDLs, in which cholesterol and phospholipids from tissues are collected and, ultimately, returned to the liver for their final deposition [15,16]. Recent evidence indicates that, besides lipid efflux, HDL deliver cholesterol and other lipids to peripheral cells [17,18,19,20]. In this context, the HDL subclasses are the result of a complex bidirectional exchange of lipids, and, consequently, the clinical meaning of these subclasses remains to be elucidated.

The clearance of HDL particles from plasma is mainly carried out by the liver, but earlier studies have suggested that the kidneys are also a major catabolic site for apo A1 [21,22]. In the same context, other studies have suggested that a fraction of lipid–poor apo A1 and, probably, small HDL are catabolized by the kidneys [21,22,23]. This observation is in line with the expression of receptors involved in the uptake of apolipoproteins in the proximal tubule epithelium [24,25] and with alterations in the relative proportion of HDL subclasses in different physiopathological circumstances, such as proteinuria and renal failure [26,27,28].

Despite previous evidence, we demonstrated that the fractional catabolic rate of HDL-apo A1 was normal in a model of rabbits with a three-quarter renal mass reduction [29]. Similarly, the kidneys modestly participate in HDL-apo A1 clearance in the proteinuria setting [27]. Therefore, the kidneys should not be regarded as important organs for HDL-apo A1 clearance, since the glomerular barrier prevents large molecular aggregates, such as lipoproteins, from being filtered into Bowman’s space. However, we observed that the HDL subclasses were significantly enriched with cholesteryl esters (CE) in the abovementioned nephrectomized rabbits [29], thus suggesting that the kidneys could be a metabolic site for cholesterol in HDL. Therefore, our aim in this study was to determine the effect of a 75% reduction in renal mass on HDL-CE turnover and its implications for HDL lipid content.

## 2. Results

The three-quarter functional renal mass reduction resulted in a significant increase in plasma creatinine and urea, whereas glucose levels remained comparable between groups (Table 1). Accordingly, creatinine clearance was lower in the nephrectomized rabbit (Ntx) group than in the sham rabbits, whereas proteinuria remained similar in both groups (Table 1).

Concerning the lipid profile, plasma total cholesterol and HDL-cholesterol increased by 60% and 35% in the Ntx group, respectively, compared with the sham group (Table 1). In particular, HDL-CE was the affected fraction, whereas HDL-free cholesterol remained comparable between groups. Triglycerides tended to decrease in the Ntx group, but the differences did not reach statistical significance. In contrast, HDL-triglyceride plasma levels in the Ntx group were significantly higher than in the sham group, while phospholipids remained comparable between groups.

**Figure 1:**
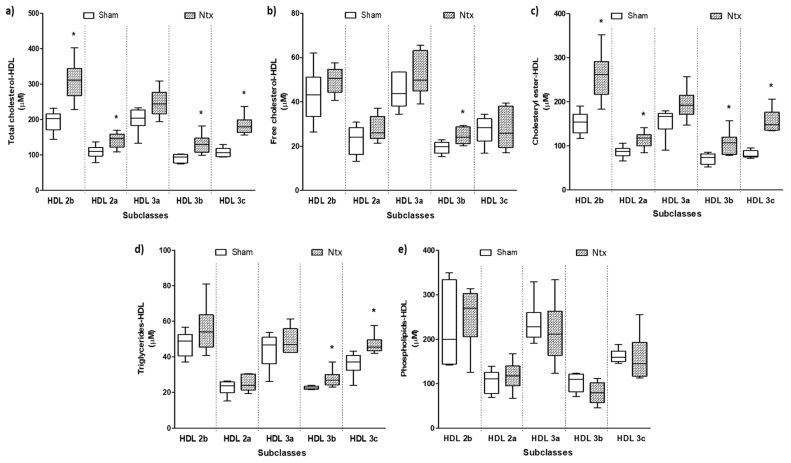
(**a**) Total cholesterol, (**b**) free cholesterol, (**c**) cholesteryl ester, (**d**) triglyceride, and (**e**) phospholipid plasma concentrations associated with each HDL subclass from Ntx and sham rabbits. HDL: high-density lipoprotein, Ntx: nephrectomized rabbits. Data are shown as median (horizontal lines) and interquartile range (boxes). *n* = 6 per group. U-Mann–Whitney test, * *p* < 0.05.

Differences in HDL-cholesterol and HDL-triglycerides between the groups suggested that there were alterations in the HDL subclasses. Accordingly, cholesterol plasma concentrations associated with the five HDL subclasses were significantly higher than those of the sham group, except for HDL 3a-cholesterol, which did not reach statistical significance (Figure 1a). This increase corresponded to the esterified cholesterol (Figure 1c) since the free cholesterol plasma levels associated with HDL subclasses remained unaltered after renal mass reduction (Figure 1b). In addition, there were slight increases in HDL 3b and 3c triglyceride plasma levels in the Ntx group compared with the sham group (Figure 1d), while there were no significant modifications in the phospholipid plasma levels (Figure 1e).

Since there is a defined number of phospholipid molecules on the surfaces of lipoproteins of a specific size, the lipid-to-phospholipid ratio is considered a marker of lipid content in HDL subclasses [30]. Therefore, we determined the lipid ratios in HDL, and the results are shown in Table 2. As expected, HDL particles were enriched with cholesterol in the Ntx group; the cholesteryl-esters-to-phospholipids ratios (CE/PH) of all the HDL subclasses tended to increase, but only those of the small HDL 3b and 3c were significantly augmented, more than doubled compared with the corresponding HDL subclasses of the sham rabbits (Table 2).

To establish the metabolic origin of cholesterol accumulation in HDL in our animal model of renal mass reduction, we determined the plasma kinetics of cholesteryl esters in vivo. We intravenously infused HDL labeled with [^3^H]-cholesteryl esters (HDL-[^3^H]-CE), and we recorded the radioactivity of the plasma (in HDL and lipoproteins containing apo B (VLDL/LDL)) in time. The removal of HDL-[^3^H]-CE in the plasma compartment of the Ntx group decreased compared with the sham group; the calculated fractional catabolic rate (FCR) was 40% lower in the Ntx group than in the sham group (0.14 (0.10–0.19) vs. 0.24 (0.17–0.36) h^−1^, respectively; *p* < 0.05). We assumed that rabbits were in a steady state during the kinetic study; thus, the calculated production rate (PR) of the HDL-cholesteryl esters was similar between groups (4.46 (3.02–6.96) vs. 5.50 (4.22–7.97) mg·kg^−1^·h^−1^ in the Ntx and sham groups, respectively; *p* = NS).

**Figure 2:**
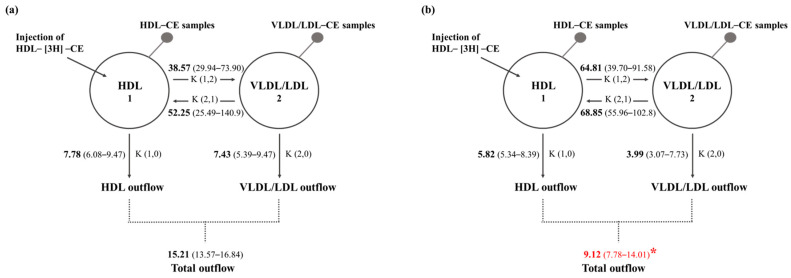
Compartmental model for the transfer of CE between lipoproteins from the (**a**) sham and (**b**) Ntx groups. HDL labeled with [^3^H]-cholesteryl esters were intravenously administered to rabbits; blood samples were collected, and HDL (compartment 1) and VLDL/LDL (compartment 2) were isolated to measure radioactivity in time (see the Methods section). The transfer of CE from HDL to VLDL/LDL is represented by the coefficient K (1,2); the transfer of CE from VLDL/LDL to HDL is represented by the coefficient K (2,1); the CE flow associated with HDL from plasma is represented by the coefficient K (1,0), and the outflow of VLDL/LDL-CE is represented by the coefficient K (2,0). Numbers represent the amount of CE transfer mass from one compartment to the other or from the plasma, expressed as μmol/L per minute (μmol·L^−1^·min^−1^) for one compartment to the other or from the plasma. Total mass outflow was calculated as the addition of HDL and VLDL/LDL cholesteryl esters removed from the plasma compartment (K (1,0) + K (2,0)). HDL: high-density lipoprotein, VLDL: very-low-density lipoprotein, LDL: low-density lipoprotein, CE: cholesteryl ester, 1: compartment 1, 2: compartment 2. Data are shown as median (interquartile range). U-Mann–Whitney test, * *p* < 0.05.

We further measured fluxes in the transfer of HDL-[^3^H]-CE into lipoproteins containing apo B (VLDL/LDL) and the outflow of [^3^H]-CE from the plasma compartment; the kinetic model for this analysis is described in the Methods section. The CE fluxes between HDL and VLDL and vice versa tended to be higher in the Ntx group, but the differences did not reach statistical significance (Figure 2 and Supplementary Table S1). Furthermore, direct CE fluxes between HDL and VLDL/LDL from the plasma compartment tended to be lower (Figure 2). In contrast, the total outflow of CE from both HDL and VLDL in the Ntx group was significantly lower than that of the sham group (9.12 (7.78–14.01) vs. 15.21 (13.57–16.84) μmol·L^−1^·min^−1^ for the Ntx and sham groups, respectively; *p* < 0.05; Supplementary Table S1).

## 3. Discussion

In this study, we demonstrated that 75% renal mass reduction resulted in an increase in HDL-cholesterol plasma levels, as cholesteryl esters are the species responsible for this increase. In addition, the FCR and the total outflow of cholesteryl esters from the plasma compartment in the Ntx group were lower than in the sham group.

Dyslipidemias are common features of several renal disorders; in particular, nephrotic syndrome, chronic kidney disease, and end-stage renal disease are characterized by decreased HDL-cholesterol plasma levels [26,28,31,32]. In contrast with these diseases, our model was characterized by a lack of significant proteinuria, which demonstrates the integrity of the glomerular ultrafiltration membrane. The functional renal mass reduction in our animal model was confirmed by increased creatinine and urea plasma levels and decreased creatinine clearance. Moreover, the functionality of the remaining 25% of glomeruli seemed to be preserved during the 4 weeks of evolution after partial nephrectomy, since proteinuria remained similar to that in the sham group, as mentioned above. A lack of increased membrane permeability was crucial for our purposes because marked proteinuria is associated with an increased HDL-apo A1 FCR in rabbits [27]. Therefore, with this animal model, we were able to explore the kidneys’ effect on HDL-cholesteryl ester kinetics without any interference from increased HDL particle renal clearance.

The cubilin and megalin receptors in proximal tubule cells participate in protein reabsorption from the glomerular filtrate, including apo A1 and HDL, among other proteins [21,24,25]. In the context of a functional glomerular filtration barrier in cubilin haploinsufficient mice, Aseem et al. demonstrated that HDL 3 clearance increased without changes in HDL 2 [24]. However, we believe that the level of HDL glomerular filtration is negligible when the integrity of the ultrafiltration membrane is preserved, as we previously demonstrated [27,29]. Consequently, the kinetic results of this study provide alternative explanations for the altered HDL subclasses associated with a reduced renal mass.

A lower FCR is the kinetic explanation for the increased plasma HDL-cholesteryl esters in Ntx rabbits. We also observed decreased VLDL/LDL and HDL cholesteryl ester mass outflow from plasma, which synergistically contributed to the decreased total cholesterol plasma levels in Ntx rabbits. This observation suggests that the tissues of Ntx rabbits require less cholesterol; since LDL and HDL deliver cholesterol to tissues [17,33], the removal of one kidney and a 50% infarct of the other in Ntx animals reduced the demand for cholesterol in these organs, decreasing the total outflow of this lipid from plasmatic lipoproteins. Congruently, HDL-cholesterol and other plasma lipids increase in kidney donors [34].

Despite the decreased cholesteryl ester FCR in Ntx rabbits, cholesteryl esters transferred between lipoproteins or out of the plasma compartment via HDLes were not significantly modified by renal mass reduction. Of note, the kinetics of non-esterified cholesterol were not considered in this study. In this context, we cannot discount the idea that the inflow of non-esterified cholesterol into HDL and ulterior esterification contributed to the accumulation of cholesteryl esters in Ntx rabbits. In these circumstances, the source of cholesterol excess was certainly the section of the kidney that was infarcted and remained inside the abdominal cavity; this tissue surplus was in the process of gradual elimination and its cholesterol was in turn recovered by HDL. This explanation is congruent with the HDL subclass modifications, i.e., increased CE and a tendency toward increased CE/PH in HDL subclasses.

Since a specific number of phospholipids defines the size of HDLs, the surface components of HDL subclasses are considered markers of the number of particles, and the lipid-to-phospholipid ratio indicates the content of these lipids in lipoproteins [30]. Considering that the phospholipids of HDL subclasses were comparable between groups in our study, it is likely that the number of HDL particles remained constant after nephrectomy. However, HDL subclasses were enriched with cholesteryl esters, particularly small HDL, which had significantly higher CE/PH ratios than in the sham rabbits. This interpretation is also congruent with the normal production rates and decreased FCR of cholesteryl esters that we observed.

In this study, the high levels of HDL-cholesterol were likely the result of a decreased demand for cholesterol due to the suppression of most of the kidney cells’ mass. With all the appropriate reserves, these observations may explain the relationship between high plasma HDL-cholesterol levels and disease progression in patients with chronic kidney disease (CKD), as has been observed in the general population [35]. Additionally, time-varying Cox analysis further supported the fact that low and high HDL-cholesterol plasma levels are related to adverse renal outcomes in patients with CKD, as recently demonstrated by a robust prospective study [36]. In agreement, higher HDL-cholesterol levels were associated with an increased risk of rapid GFR decline in a general non-diabetic population after a follow-up of 5.6 years [37]. In light of the present study, we believe that high HDL-cholesterol is not a cause of renal damage progression. Instead, high HDL-cholesterol levels may simply be a biomarker of accelerated renal mass loss, which, in turn, is the cause of steeper GFR decline rates; a lower number of cells demanding lipids from HDL induce its accumulation in plasma. More complete characterizations of HDL subclasses in CKD patients will help to discern the significance of high HDL-C levels in renal disease progression.

In summary, in this study, 75% of renal mass reduction, absent of proteinuria, was characterized by increased cholesteryl esters in HDL subclasses, a normal PR, and a decreased FCR for HDL-cholesteryl esters. The transfer of cholesteryl esters between lipoproteins in the plasma compartment was normal, whereas small HDL subclasses were enriched with cholesterol. The overall kinetics of HDL-cholesteryl esters suggest decreased lipid delivery to tissues, which is probably associated with a reduced demand for cholesterol associated with reductions in renal mass. These results reinforce the idea that the kidneys do not play a role in cholesterol metabolism; instead, the kidneys require cholesterol from plasma HDL and other lipoproteins, and, consequently, the renal mass decrease is associated with higher levels of cholesterol in these lipoproteins.

## 4. Materials and Methods

### 4.1. Animals

Male New Zealand white rabbits weighing 3.0 to 3.5 kg were randomized into nephrectomized (Ntx) and sham groups. For nephrectomy, two branches of the left renal artery were infarcted via ligation during the initial surgical procedure. Two weeks later, a second surgery was performed to completely remove the right kidney while preserving the adrenal gland. Consequently, only 25% of the functional renal mass was preserved in this animal model. Simulated surgical procedures were performed on the sham control group for comparison. Animals had free access to a standard chow diet and water ad libitum. HDL-cholesteryl ester (HDL-CE) kinetic experiments were performed after four weeks of evolution of the second surgery.

All procedures on rabbits complied with the guidelines of the “Guide for the Care and Use of Laboratory Animals” [38], the Mexican Federal Regulation for animal experimentation and care (NOM-062-ZOO-1999), and were approved by the Institutional Animal Care and Use Committee of the Instituto Nacional de Cardiología Ignacio Chávez, registration number INC/CICUAL/PIL/005/2023.

### 4.2. Sample Collection

Twelve-hour fasting blood samples were drawn from the central artery of the ear through tubes with sodium heparin (15 UI/mL) as an anticoagulant. Blood samples were centrifuged for 15 min at 1300× *g*. The plasma was separated and frozen at −70 °C until use. Animals were placed in metabolic cages to collect 24 h urine for the determination of creatinine, urea, proteinuria, and creatinine clearance.

### 4.3. Biochemical Analyses

The total cholesterol, triglyceride, glucose, creatinine, urea, and urine protein concentrations were determined via enzymatic colorimetric methods (Randox Laboratories, Antrim, UK). The phosphotungstic acid–Mg^2+^ method (Randox Laboratories, Antrim, UK) was used to precipitate non-HDL lipoproteins in the plasma; HDL-lipids (total cholesterol, free cholesterol, triglycerides, and phospholipids) were determined in the supernatant fraction via enzymatic colorimetric methods (Randox Laboratories, Antrim, UK and Wako Chemicals, Richmond, VA, USA). Cholesteryl esters were calculated as the difference between total and free cholesterol expressed in mmol/L. Biochemistry assessments were performed in post-surgical conditions. Lipids of apo B-containing lipoproteins were determined in ultracentrifuged samples, as previously described [39].

### 4.4. Isolation and Characterization of HDL Particles

HDL (δ 1.21 g/mL) were isolated via ultracentrifugation using KBr to adjust the plasma density, as previously described [39]. Isolated HDL were dialyzed against 0.09 M of Tris/0.08 M boric acid/3 mM EDTA buffer, pH 8.4, or 8.6 mM of Na_2_HPO_4_/1.4 mM NaH_2_PO_4_/150 mM NaCl buffer (PBS), pH 7.4 (PBS), for kinetic studies.

To analyze HDL subclasses, 25 μg of HDL-protein was separated over 22 h at 180 V in 3–30% polyacrylamide gradient gels under native conditions. Gels were stained for total cholesterol, free cholesterol, phospholipids, and triglycerides using non-commercially available enzymatic mixtures developed by our group, as previously described [29,30]. Densitograms of stained lipids and proteins were used to determine HDL subclasses considering the following size intervals: HDL 3c, 7.94–8.45 nm; HDL 3b, 8.45–8.98 nm; HDL 3a, 8.98–9.94 nm; HDL 2a, 9.94–10.58 nm; and HDL 2b, 10.58–13.59 nm [29,30]. A high-molecular-weight calibration kit for globular proteins (Amersham Pharmacia Biotech, Buckinghamshire, UK) was used as a reference for hydrodynamic size [29,30]. The relative proportion of lipids in each HDL subclass was defined as the percentage of the area under the curve of each size interval with respect to the total area under the curve determined via densitometric analysis. The plasma concentrations of the total cholesterol, free cholesterol, triglycerides, and phospholipids of each subclass were calculated as previously reported [29,30].

### 4.5. Preparation of HDL-Containing [^3^H] Labeling Cholesteryl Esters and Turnover Studies

Lipoproteins containing apo B (VLDL/LDL) were removed from plasma via ultracentrifugation, using KBr to adjust the density to δ 1.063 g/mL. The infranatant fraction was dialyzed against a PBS buffer and incubated with 1 µCi/mL of 1α,2α-^3^H-cholesterol ([^3^H]C) in ethanolic solution (tritiated cholesterol, American Radiolabeled Chemicals Inc., St. Louis, MO, USA) at 37 °C for 18 h, as previously described [39]. With this method, more than 90% of the [^3^H]C was incorporated into the HDL and esterified via intrinsic lecithin–cholesterol acyltransferase.

HDL-[^3^H]-cholesteryl esters (-CE) were isolated via ultracentrifugation and dialyzed against PBS. [^3^H]C unincorporated into HDL was removed via size exclusion chromatography (Sephadex G-25 medium), using PBS as a mobile phase. The eluted fractions containing HDL-[^3^H]-CE were pooled, sterilized using 0.22 µm filters (Millipore, Burlington, MA, USA), and stored at 4 °C until use.

### 4.6. In Vivo HDL-[^3^H]-CE Kinetic Studies

Rabbits received an intravenous bolus containing 1 × 10^6^ counts per minute (cpm) of HDL-[^3^H]-CE in a total volume of 0.5 mL. Blood samples (1.0 mL) were obtained from the opposite marginal vein 5 min after administration and then at 15, 30, 45, 60, 90, 120, 150, 180, 240, and 300 min. Plasma (300 µL) was ultra-centrifugated at δ 1.063 g/mL. The supernatant containing the VLDL/LDL fraction was recovered, and radioactivity was measured using a liquid scintillation analyzer (TRI-CARB 2200CA; Packard Instruments, Downers Grove, IL, USA). HDL were extracted from the infranatant fraction via ultracentrifugation at δ 1.25 g/mL, and radioactivity was measured. The radioactivity kinetic curves of [^3^H]-CE were constructed by considering the cpm at 5 min as 100% and 0% for the HDL and VLDL/LDL fractions, respectively.

### 4.7. Compartmental Modeling

The fractional catabolic rate (FCR) of the cholesteryl esters was computed by fitting radioactivity kinetics percentages to a biexponential function using the SAAMII software v1.1 (SAAM Institute, Seattle, WA, USA). The cholesteryl ester production rate (PR) was determined using the following formula: [PR = FCR × CE plasma concentration (mg/L) × total plasma volume (L)/body weight (kg)] [40]. The plasma volume was assumed to be 0.0328 L/kg of body weight [41].

**Figure 3:**
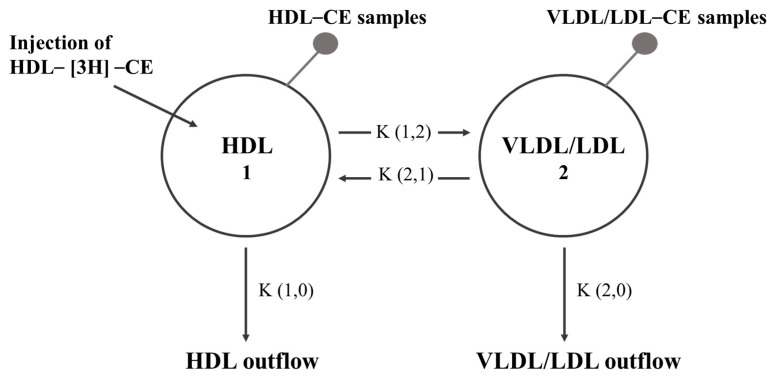
Compartmental model for the rate of mass transfer of CE between lipoproteins. Circles represent HDL and VLDL/LDL, identified as compartments 1 and 2, respectively. Arrows represent the direction of transfer or the elimination pathway of CE. Small gray circles represent the compartment where the samples were obtained for the radioactivity count. The transfer of CE from HDL to VLDL/LDL is represented by the coefficient K (1,2); the transfer of CE from VLDL/LDL to HDL is represented by the coefficient K (2,1); the flow of CE associated with HDL out of the plasma is represented by the coefficient K (1,0); and the outflow of VLDL/LDL-CE is represented by the coefficient K (2,0). HDL: high-density lipoproteins, VLDL: very-low-density lipoprotein, LDL: low-density lipoprotein, CE: cholesteryl ester.

The specific mass transference of CE (μmol·L^−1^·min^−1^) between lipoproteins and their direct removal from the plasma compartment were evaluated with a two-compartment model (Figure 3), as previously described [39]. Briefly, two assumptions were made: (1) given that the concentration of CE from VLDL was very low, the VLDL/LDL fraction was treated as a single pool; (2) the entry of CE into the system was exclusively through the HDL-[^3^H]-CE pool. In this way, compartment 1 corresponded to the HDL fraction, while compartment 2 constituted the VLDL/LDL fraction. Four transfer coefficients were considered in the model: coefficient (1,2) represented the transfer of CE from HDL to VLDL/LDL; coefficient (2,1) represented the transfer of CE from VLDL/LDL back to HDL; and coefficients (1,0) and (2,0) represented the direct removal of CE from the HDL and VLDL/LDL plasma compartments, respectively.

### 4.8. Statistical Analysis

Data are expressed as the median (interquartile range). Comparisons between groups were made using the U Mann–Whitney test. The significance of differences was set at * *p* < 0.05.

## Figures and Tables

**Table 1:** Post-surgery biochemistry characteristics.

	Sham (*n* = 6)	Ntx (*n* = 6)
Total cholesterol (mmol/L)	1.18 (0.86–1.39)	1.89 (1.42–2.04) *
Triglycerides (mmol/L)	1.19 (1.0–1.32)	0.99 (0.80–1.18)
HDL-cholesterol (mmol/L)	0.74 (0.67–0.78)	1.00 (0.78–1.14) *
HDL-free cholesterol (mmol/L)	0.15 (0.13–0.17)	0.17 (0.16–0.22)
HDL-cholesteryl esters (mmol/L)	0.59 (0.50–0.63)	0.80 (0.62–1.04) *
HDL-triglycerides (mmol/L)	0.18 (0.16–0.19)	0.23 (0.19–0.26) *
HDL-phospholipids (mmol/L)	0.84 (0.63–1.08)	0.89 (0.85–1.50)
Non-HDL-cholesterol (mmol/L)	0.45 (0.12–0.73)	0.92 (0.70–1.64) *
Glucose (mmol/L)	7.40 (7.03–7.63)	6.89 (6.76–7.00)
Urea (mg/dL)	40.22 (35.87–45.49)	60.85 (56.67–71.69) *
Creatinine (mg/dL)	2.06 (1.95–2.10)	2.98 (2.61–3.34) *
Creatinine clearance (mL/min)	4.29 (3.19–4.59)	1.55 (1.26–2.23) *
Proteinuria (g/24 h)	0.13 (0.10–0.28)	0.19 (0.15–0.26)

HDL: high-density lipoproteins; Ntx: nephrectomized rabbits. Data are shown as median (interquartile range). U-Mann–Whitney tests, * *p* < 0.05.

**Table 2:** Lipid plasma concentration ratios of HDL subclasses.

Lipid	Subclasses				
	HDL 2b	HDL 2a	HDL 3a	HDL 3b	HDL 3c
TC/PH Sham	1.11 (0.53–1.31)	1–09 (0.85–1.45)	0.88 (0.63–1.06)	0.84 (0.66–1.25)	0.63 (0.58–0.77)
TC/PH Ntx	1.18 (0.96–1.63)	1.21 (0.89–1.65)	1.15 (0.87–1.62)	1.72 (1.27–2.39) *	1.19 (0.91–1.66) *
FC/PH Sham	0.22 (0.15–0.27)	0.25 (0.16–0.28)	0.19 (0.14–0.25)	0.18 (0.15–0.22)	0.16 (0.14–0.20)
FC/PH Ntx	0.18 (0.16–0.26)	0.21 (0.19–0.31)	0.22 (0.20–0.32)	0.31 (0.23–0.48) *	0.20 (0.09–0.30)
CE/PH Sham	0.89 (0.38–1.04)	0.83 (0.70–1.13)	0.72 (0.46–0.81)	0.66 (0.50–1.04)	0.48 (0.43–0.58)
CE/PH Ntx	1.01 (0.77–1.39)	1.00 (0.70–1.34)	0.94 (0.66–1.30)	1.47 (0.94–1.91) *	0.99 (0.81–1.33) *
TG/PH Sham	0.25 (0.16–0.28)	0.22 (0.19–0.31)	0.18 (0.15–0.23)	0.21 (0.18–0.28)	0.23 (0.20–0.25)
TG/PH Ntx	0.21 (0.17–0.33)	0.20 (0.16–0.33)	0.22 (0.17–0.35)	0.33 (0.26–0.60)	0.33 (0.24–0.41)

HDL: high-density lipoprotein; Ntx: nephrectomized rabbits. TC: total cholesterol; PH: phospholipid; FC: free cholesterol; CE: cholesteryl ester; TG: triglyceride. Data are shown as median (interquartile range). *n* = 6 per group. U-Mann–Whitney tests, * *p* < 0.05.

## Data Availability

The data presented in this study are available upon request from the corresponding authors.

## Supplementary Materials

The following supporting information can be downloaded at: https://www.mdpi.com/article/10.3390/ijms242317090/s1.
