# Supplementary material for: Total Outflow of High-Density Lipoprotein–Cholesteryl Esters from Plasma Is Decreased in a Model of 3/4 Renal Mass Reduction
Source: Int J Mol Sci. 2023 Dec 4;24(23):17090. doi: 10.3390/ijms242317090 (PMC10707367; doi:10.3390/ijms242317090)
Supplement: Supplementary file 1 [file ijms-24-17090-s001.zip › ijms-2721678-supplementary.pdf]

Supplementary Table S1. Transfer rates of cholesteryl esters ($\mu mol \cdot L^{-1} \cdot min^{-1}$) between lipoproteins and total outflow of cholesteryl esters from plasma compartment.

Constants	Transfer direction of CE	Sham (n = 6)	Nx (n = 6)	p-value
K (1,2)	HDL to VLDL/LDL	38.57 (29.94-73.90)	64.81 (39.70-91.58)	0.25
K (2,1)	VLDL/LDL to HDL	52.25 (25.49-140.9)	68.85 (55.96-102.8)	0.43
K (1,0)	HDL CE removal (direct pathway)	7.78 (6.08-9.47)	5.82 (5.34-8.39)	0.07
K (2,0)	VLDL/LDL CE removal (indirect pathway)	7.43 (5.39-9.47)	3.99 (3.07-7.73)	0.11
Total CE mass outflow	(HDL-CE + VLDL/LDL-CE) removal	15.21 (13.57-16.84)	9.12 (7.78-14.01)	0.03

HDL: high-density lipoproteins, VLDL: very low-density lipoproteins, LDL: low-density lipoprotein, CE: cholesteryl esters. Data are shown as median (interquartile range). Total mass outflow was calculated as the addition of HDL and VLDL/LDL cholesteryl esters removed from out of the plasma compartment U-Mann Whitney test.
